# Categorizing biomedicine images using novel image features and sparse coding representation

**DOI:** 10.1186/1755-8794-6-S3-S8

**Published:** 2013-11-11

**Authors:** Jianqiang Sheng, Songhua Xu, Xiaonan Luo

**Affiliations:** 1 National Engineering Research Center of Digital Life, State-Province Joint Laboratory of Digital Home Interactive Applications, School of Information Science & Technology, Sun Yat-sen University, Guangzhou, 510006, P. R. China; 2 Department of Information Systems, College of Computing Sciences, New Jersey Institute of Technology, University Heights, Newark, NJ, 07102, USA

## Abstract

**Background:**

Images embedded in biomedical publications carry rich information that often concisely summarize key hypotheses adopted, methods employed, or results obtained in a published study. Therefore, they offer valuable clues for understanding main content in a biomedical publication. Prior studies have pointed out the potential of mining images embedded in biomedical publications for automatically understanding and retrieving such images' associated source documents. Within the broad area of biomedical image processing, categorizing biomedical images is a fundamental step for building many advanced image analysis, retrieval, and mining applications. Similar to any automatic categorization effort, discriminative image features can provide the most crucial aid in the process.

**Method:**

We observe that many images embedded in biomedical publications carry versatile annotation text. Based on the locations of and the spatial relationships between these text elements in an image, we thus propose some novel image features for image categorization purpose, which quantitatively characterize the spatial positions and distributions of text elements inside a biomedical image. We further adopt a sparse coding representation (SCR) based technique to categorize images embedded in biomedical publications by leveraging our newly proposed image features.

**Results:**

we randomly selected 990 images of the JPG format for use in our experiments where 310 images were used as training samples and the rest were used as the testing cases. We first segmented 310 sample images following the our proposed procedure. This step produced a total of 1035 sub-images. We then manually labeled all these sub-images according to the two-level hierarchical image taxonomy proposed by [1]. Among our annotation results, 316 are microscopy images, 126 are gel electrophoresis images, 135 are line charts, 156 are bar charts, 52 are spot charts, 25 are tables, 70 are flow charts, and the remaining 155 images are of the type "others". A serial of experimental results are obtained. Firstly, each image categorizing results is presented, and next image categorizing performance indexes such as precision, recall, F-score, are all listed. Different features which include conventional image features and our proposed novel features indicate different categorizing performance, and the results are demonstrated. Thirdly, we conduct an accuracy comparison between support vector machine classification method and our proposed sparse representation classification method. At last, our proposed approach is compared with three peer classification method and experimental results verify our impressively improved performance.

**Conclusions:**

Compared with conventional image features that do not exploit characteristics regarding text positions and distributions inside images embedded in biomedical publications, our proposed image features coupled with the SR based representation model exhibit superior performance for classifying biomedical images as demonstrated in our comparative benchmark study.

## Introduction

The literature in the broader biomedical domain presents abundant image content. For example, a significant number of biomedical articles carry multiple images or graphs in their main text. Popular types of embedded image content include microscopy images, gel electrophoresis images, graphical tables, diagrams, and charts, which are adopted for visually communicating the key research ideas pursued, main theses argued, primary experimental results produced, and central findings derived in a study. Compared to their text counterpart, images and graphs carried in biomedical articles can greatly facilitate the intuitive grasp of an article's main content through quick browsing and navigation--a popular practice by many researchers in reality to cope with the exploding volume of the literature published in their fields at an ever accelerating rate. In addition to being able to summarize and highlight key content of a study, an image can also report a comprehensive set of results beyond the scope of discussion by text in a paper, e.g. the use of heatmap images to report hundreds or thousands of records of experimental results simultaneously at one place. For both types of communication purposes, an image is indeed worth a thousand words.

Despite the tremendous importance of images and graphs in biomedical publications, previously only limited efforts have been dedicated to mining such rich graphical content, in contrast to the counterpart problem of mining text content in the biomedical literature where an overwhelmingly abundant body of studies have been contributed [2-4]. Fortunately, mining of images in the biomedical literature has started receiving more research attention recently [5-8]. Part of this emerging research interest in mining biomedical images is fostered by the free availability of large-scale image repositories to the general public. For example, PMC [9] is a public database archive provided by National Library of Medicine under the U.S. National Institutes of Health (NIH/NLM). PMC offers the full text of more than 2.8 million articles in biomedical and life sciences, including all images embedded in these articles.

Two important problems of biomedical image mining are: 1) extracting image features to characterize the content of a biomedical image and 2) biomedical image categorization, both of which can help computer gain deeper understanding into the content of an image. Overall, enhancing automatic image content understanding can benefit a collection of applications in biomedical image processing, such as content-based image retrieval, recommendation, thematic topic detection, mining for trend discovery, as well as image-based or facilitated biomedical literature retrieval, navigation, content clustering, topic extraction, and literature mining-based knowledge discovery. It is noted that the image content characterizing features can directly help categorize biomedical images more accurately and reliably; while the machine learning procedure deployed at the heart of an image categorization method can also work with other image features for accomplishing the same goal of image categorization as well as other high-level, semantically-oriented image processing tasks that involve machine learning techniques. Therefore, the algorithmic advancements in overcoming the two problems are mutually supportive.

Traditional image features, such as texture, color, and shape-based features, only offer limited discriminative power for characterizing the content of biomedical images; while current general-purpose image classification methods do not achieve satisfactory precision in dealing with biomedical images. Recognizing the technical limitations of the-state-of-the-art regarding the above two important problems, we conducted this study. To address the first problem of designing effective features for characterizing biomedical images, we propose a set of novel image features that quantitatively explore and exploit the spatial distributions of text elements appearing inside a biomedical image. To the best of our knowledge, none of these features has been previously studied in the biomedical image literature. To address the second problem of introducing advanced categorization methods for biomedical images, we propose an improved Sparse Coding Representation (SCR) based technique to classify biomedical images using our newly proposed image features. By using the novel image features and the improved SCR-based classification method both introduced at this paper, we can categorize biomedical images with better accuracy and reliability than the peer state-of-the-art practice, the conclusion of which shall be verified through the experimental results reported later in this paper.

The rest of the paper is organized as follows. We first briefly review some work closely related to this study. Next, we present the novel image features that we propose for characterizing the categories of biomedical images. To extract these image features, we then introduce some algorithmic pre-processing procedures. In the next, we present our improved SCR method and apply the method for categorizing biomedical images using the novel image features introduced earlier. To explore the effectiveness of the newly proposed image features and the improved SCR method based image categorization approach using these features, we report results of benchmark experiments that measure and compare the performance of the new approach with that of the state-of-the-art peer practice. After presenting the positive comparative experimental results confirming the advantages of the new biomedical image features and the companion categorization method for biomedical images using these features, we conclude the paper in the end.

## Related work

In this section, we will briefly overview some previous studies that closely relate to our work here, including the design of image features and methods for image categorization.

### Image features

A fundamental problem of image processing is to represent an image's content in a way that automatic computer algorithms or programs can understand the representation. For this purpose, image features are often leveraged to derive machine-readable representations of image content. To design and extract image features, many strategies have been previously explored for multiple image-related application fields. On the lowest level, pixel values in an image provide some direct, low-level features for characterizing an image's content. For example, Kim et al. [10] exploited distributions of pixel values of sub-regions in an image as features for image scaling operations. They provided a novel scaling algorithm, called "winscale," which uses a maximum of four pixels from an original image to calculate a counterpart pixel in a scaled-down version of the image. Above the pixel-level features, Jebara [11] proposed image features based on bags-of-pixels for modeling related visual objects in an image. For example, they modeled gray scale images as a bag of pixel vectors. This practice means a permutational invariance over the features of bag of pixels, which is actually processed by describing each image with a permutation matrix. Over the past serval years, many researchers also explored visual features of color for image application. Swain [12] developed a technique that matches color-space histograms for recognizing objects through color indexing. They provided a term of *Histogram Intersection*, which allows real-time indexing in a large multicolored image database. Stricker [13] also used the color to conduct image indexing. The innovative aspect of their method lies in the design where the method stores the first three moments of each color channel in an image rather than stores the complete color distributions in the image. Such treatment focuses on capturing the dominant color elements in an image. Gervers et al. [14] used color features to recognize visual objects. Their approach works particularly well to robustly recognize color objects that undergo substantial changes in viewpoints, geometries, and illumination conditions. People have also observed texture patterns in an image as formulated by structural distributions of pixel values in the image. Such type of texture based image features also work at a higher level than pixel value based image features to characterize image content for visual object recognition and image regions of interest identification. Along this line of research, Haralick et al. [15] exploited image textural features for image classification. Weyand et al. [16] utilized global textural features of an image for image retrieval. Another type of image features popularly adopted in existing work is image edges, e.g. the edge analysis based algorithm for medical image segmentation [17]. Among all edge-based image feature extraction methods, SIFT [18] and SURF [19] features are most eminently recognized. For example, Ledwich and Williams [18] introduced a method that reduces the size and complexity of SIFT features for image retrieval. Yi et al. [20] matched SIFT features for multi-spectral remote image registration. Wojnar and Pinheiro [19] presented a method for annotating medical images through using an image's SURF descriptor, the method of which can significantly improve the classification accuracy of lung images. Wang et al. [21] also leveraged SURF features to develop a non-rigid method for robust and efficient registration of medical images. Their experimental results showed that their new SURF feature based registration method performs much faster and more robustly than conventional image registration approaches.

Recently, people have proposed many semantic-oriented, high-level image features for image content representation, e.g. extracting features from image regions around points of interests [5,6] as well as a variety of bag-of-features [22,23]. Cao et al. [24] designed a new class of bag-of-features for addressing the particular application needs of large-scale image retrieval. Their method proposed spatial bag-of-features by projecting ordered bag-of-features from multiple directions or points. Yanai [25] employed a region-based bag-of-features representation and the multiple instance learning method to implement a novel image gathering system. Using these region-based features, their method can more satisfyingly separate foreground image regions from background regions for effectively deriving image training data. Based on traditional bag of words, Garg [26] explain this from a soft computing perspective. Their results revealed that this fuzzy and possibilistic codeword assignment significantly boosted the image classification accuracy. In biomedical image application aspect, for instance, Tommasi et al. [22] annotated medical images using bags-of-features (BoF). In their work, medical images are represented both by global and local descriptions. Rafkind et al. [27] introduced a biomedical image categorization method that integrates image caption text with its intensity histograms, edge-direction histograms, and edge-based axis features. Shatkay et al. [5] introduced an approach for biomedical image categorization that uses image features based on gray-level histogram statistics, edge direction histograms, as well as the image's associated source article's abstract and full text. The most similar method to our work here is probably the hierarchical image classification method proposed by Kim and Yu [6], who explored and analyzed image features strongly associated with each type of images and developed a hierarchical image classification approach for categorizing an arbitrary image into one of the five popular types--gel images, images-of-things, images of graphs, images of models, and images of mixed content. According to image textural features, their method first separates all candidate images into two broad groups, including texture rich images and texture sparse images. For images falling into the first group, the method further examines features based on image entropy, skewness, and uniformity; for images categorized into the second group, the method analyzes features based on image edge differences, uniformity, and smoothness. After performing the second-stage feature analysis, each image is finally classified into a specific image type from among the five candidate types. The recall of their hierarchical image categorization method is superior to its predecessor methods because of the high accuracy of the first stage image categorization operation. To our best knowledge, text distribution patterns in an image have not been previously explored as content-revealing image features for categorizing biomedical images, which typically carry inside abundant embedded text.

### Image categorizing methods

A few collection of prior efforts has been dedicated to the specific topic of biomedical image categorization. Lehmann et al. [7] proposed a method for automatically categorizing medical images into more than 80 categories where previous approaches can only distinguish up to 10 categories of medical images. Giuld et al. [28] explored an approach for automate medical image categorization by using the optional tags provided by the DICOM 3.0 imaging protocol to store indicative information regarding the modality and specific regions in a medical image. Medical informatics researchers also studied how to classify images for assisting clinical diagnosis. For example, Zhang et. al. [29] designed an auxiliary tool for analyzing functional magnetic resonance images, which provides a number of image classification methods for examining 3D brain images. Balasubramanyam et al. [30] trained support vector machines (SVMs) to categorize images about healthy joints from unhealthy ones. From the methodology's perspective, in the early years, nearest neighbor-based approaches were widely used, for example by Weyand et al. in [16], for medical image classification and retrieval. Alternative methods that also receive plenty of attention include discriminative approaches such as log-linear models [31] and decision trees [32]. Later, SVM-based methods acquired more popularity because of their superior performance to the traditional nearest neighbor-based approaches. For example, Tommasi et al. [23] presented a SVM-based approach for medical image annotation, which is a problem directly related to image categorization where the produced image annotation tags can be used as essential clues for categorizing images. Similarly, Avni et al. [33] exploited a SVM-based approach to medical image retrieval and annotation. Recently, the sparse representation method attracted wide attention among the image classification field, e.g. [34-37]. Wright et al. [34] viewed the face recognition task as an image classification problem, for which they deployed the sparse representation method in combination with multiple linear regression models to obtain robust face recognition results through facial image categorization. To perform image discrimination and texture segmentation, Mairal et al. [36] introduced a cost function for a sparse representation-based classification method, which can also be directly applied for robust image categorization. Zuo and Zhang [37] proposed a sparse representation based algorithm for general-purpose image classification. Their algorithm considers both intra-class variations and background clutter among candidate images. Experimental results demonstrated that even without performing time-consuming parameter optimization, their sparse representation based method can readily achieve superior performance to the traditional methods.

Besides sparse representation-based approaches for image classification, many other classification approaches have also been explored in the research community. For example, Zou et al. [38] put forward a structure-based neural network with a back propagation through structure algorithm for classifying high-resolution remote sensing images. Experiment results show that the method provides a viable solution for classifying high-resolution panchromatic remote sensing data. Hou et al. [39] presented a new method based on manifold learning for hyperspectral image classification. Genetic programming (GP) is another popular choice for engineering image classification algorithms. Li et al. [40] adopted the GP methodology for multi-image classification in complicated application scenarios with a satisfying accuracy. Tseng et al [41] suggested that images can be classified in two ways: i) classifying according to the main objects included in an image, and ii) classifying by the relationships between multiple objects; while a large number of existing image classification methods only work with one of the above two classification criteria. To address this overlook, they proposed a hybrid image classification approach that leverages both ways of classification analysis. In [42], Wu et al. introduced a novel visual language modeling method for content-based image classification. Their method transforms each image into a matrix of visual words and assumes that each visual word is conditionally dependent on its neighbors. The new method also subtly exploited the spatial correlation between multiple visual words for the image classification purpose.

In this work, we exploit an improved version of the sparse representation method and apply the method in combination of our proposed novel image features for categorizing biomedical images. Our comparative experimental results show that the new approach achieves better performance than existing state-of-the-art practice.

## Our method

**Figure 1:**
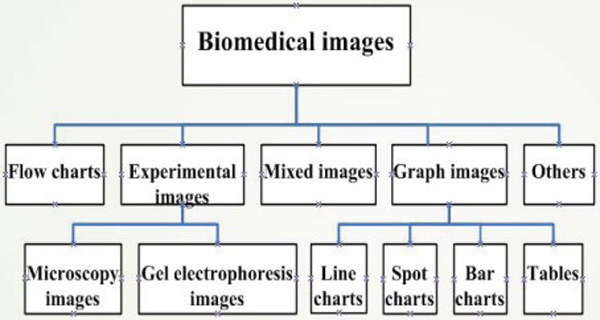
**Image taxonomy**. Taxonomy employed in our work for images embedded in biomedical publications.

In this section, we will look at our novel image features for characterizing the category of a biomedical image. In addition to the newly designed features, we will further present a new image categorization method using these new features. To categorize a biomedical image, we adopt the taxonomy for biomedical images introduced in our previous work [1]. In this taxonomy, biomedical images were divided into five main categories, which are further divided into eight sub-categories. Figure 1 illustrates this taxonomy graphically.

### Multi-panel image segmentation as a pre-processing step

Before we define the new image features and introduce their extraction method, we would like to first discuss a pre-processing task of multi-panel image segmentation, without which the reliability of the extracted image features could be severely compromised. This pre-processing step is particularly introduced to cope with images that might belong to the category of *mixed images *in our image taxonomy. When we design our image feature extraction method initially, we did not realize the need of special treatment for the mixed image category. From our experimental results, we then noticed that this category of images results in much lower image categorization performance than other categories of images. A closer analysis showed that when an image contains multiple panels or sub-images, which is a necessary but not sufficient condition for the image to be recognized as of the mixed category, the margins and non-uniform distribution of text elements among and across the image's multiple panels or sub-images could lead to ambiguity in our text distribution based image feature representation. To overcome this issue, we therefore introduce the multi-panel image segmentation procedure as a pre-processing step for our method.

**Figure 2:**
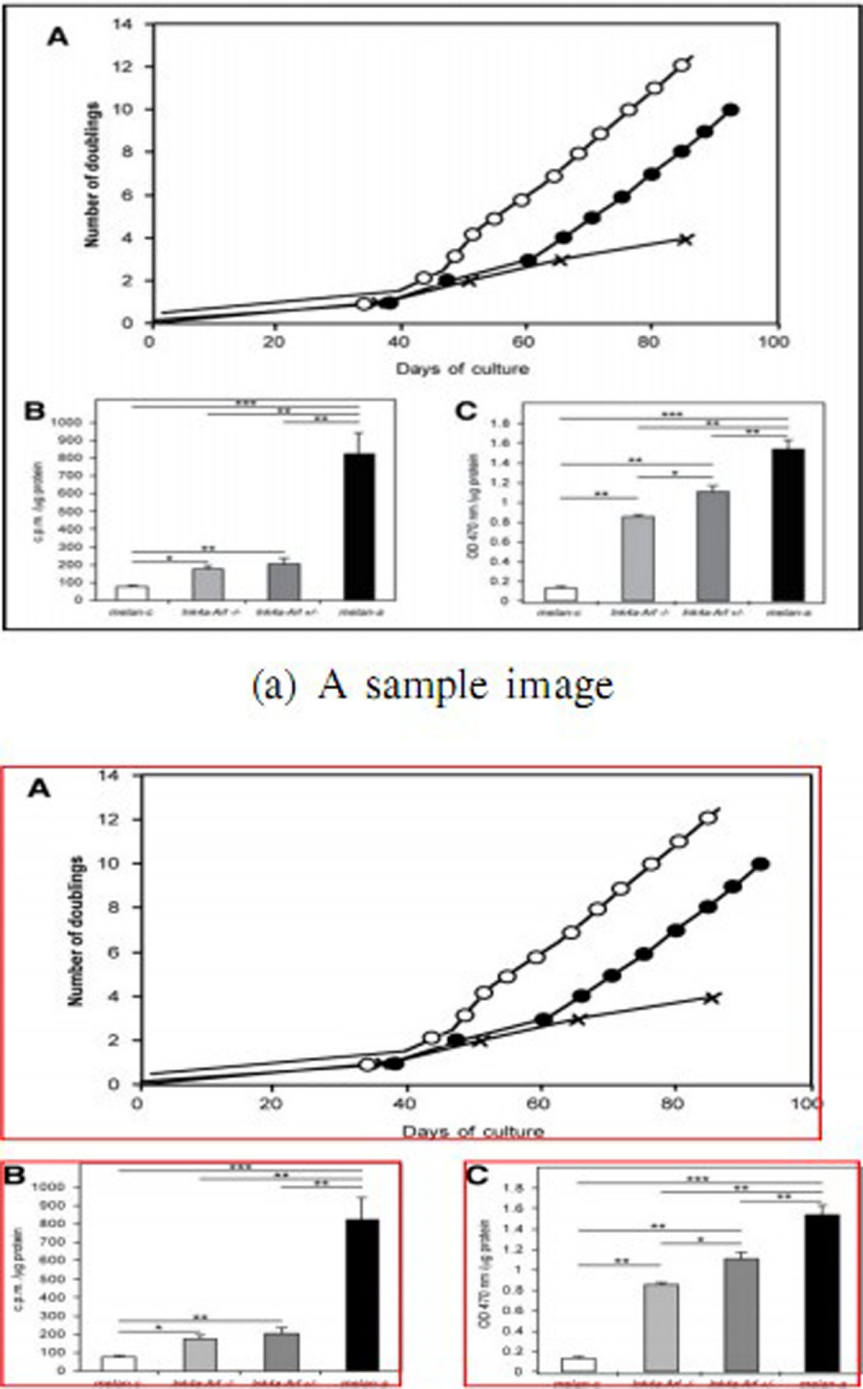
**Image segmentation result by our implemented method**. (a) A sample image from the aritlce [48], (b) sub-images decomposed from the sample image, which consists of a line chart and a bar chart.

Algorithm 1 lists the image segmentation procedure used in our work, whose main idea is as follows: We first apply the Gaussian filter function, whose implementation is offered by the OpenCV 2.2.0 package, to remove local noise in an input image *I*. We then convert the image into its binary counterpart representation. After that we scan the whole image *I *following all the horizontal and vertical scanlines in the image respectively, one scanline at a time. The goal is to find suitable horizontal and/or vertical scanlines that can segment the image *I *into its constituent panels or sub-images. For each scanline we consider, we calculate the number of foreground pixels *N_p _*in the image that lie on the line. When the number of *N_p _*is larger 5, we empirically regard the line as a candidate image segmentation line. After all the candidate image segmentation scanlines are detected, we then apply them collectively to divide *I *into multiple sub-regions. At last, we retain those divided sub-regions whose respective areas are no smaller than 1/20 of the total image area. Figure 2 shows an example image segmentation result generated by the above procedure.

Algorithm 1 Our image segmentation algorithm

Input:

**     A unprocess image ***I*

   Output:

**     Segmented image ***I_s_*

  1: convert *I *into a binary image;

  2: Derive the number of foreground pixels *N_p _*resting on each horizontal(vertical) scanning line in *I*;

  3: **if ***N_p _*≤ 5 pixels **then**

  4:        add scanning line into candidate horizontal(vertical) segment line set;

  5: **end if**

  6: *I *is segmented into grid cell regions by candidate segment lines, i.e. **S **= {*S_i_*};

  7: Calculate their area *Area*(**S**) and *Area*(*S_i_*);

  8: **if **$Area\left(S_{i}\right)\geq \frac{1}{20}Area\left(S\right)$**then**

  9:        consider *Area*(*S_i_*) as a valid segmentation region;

  10: **end if**

  11: **return**

### Novel biomedical image features

**Figure 3:**
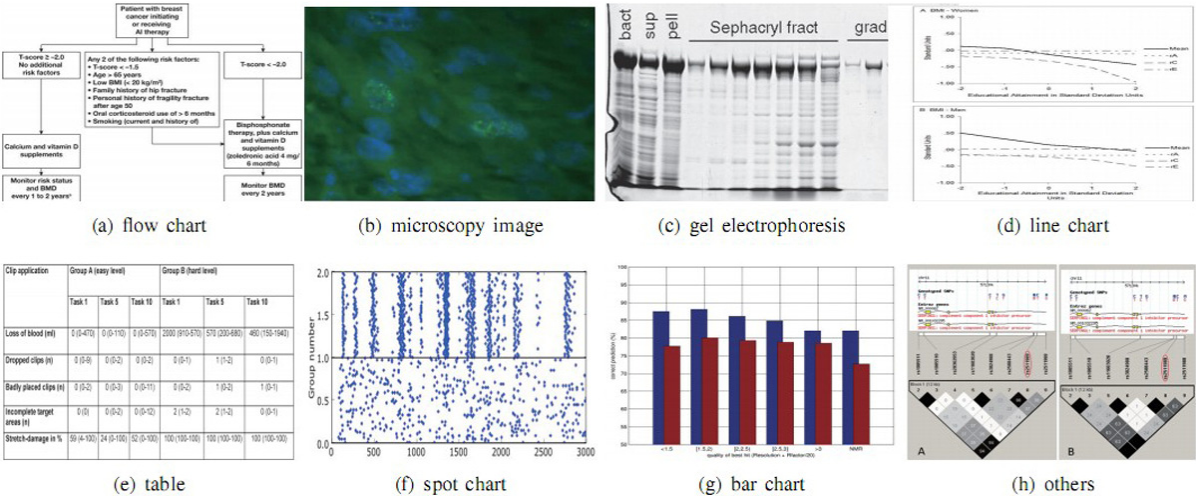
**Eight examples of image classes used in this paper**. Eight image classes and sub-classes in our image taxonomy, which are organized as a two-level class hierarchy. On the top level, images are categorized into the classes of flow charts, experimental images, graph images, mix images, and others. On the bottom level, images are further divided into eight categories where the class of experimental images is categorized into microscopy and gel electrophoresis images; the class of graph images into line charts, bar charts, spot charts, and tables.

It is easy to notice that many biomedical images contain some highly complex textural patterns or image background; in addition, visual objects displayed in a biomedical image can show low image contrast (see (a), (c), (d) in Figure 3 for examples). These visual characteristics of biomedical images render major challenges for image content understanding and categorization using traditional pixel, texture, or edge-based image features. Fortunately, as mentioned at the beginning of this paper, biomedical images possess a salient content composition property that distinguishes themselves from images in other application domains such as personal photos taken by digital cameras--the majority of biomedical images carry abundant embedded text, which is introduced either for annotating other visual objects in an image or as a primary source of content elements by itself. This image composition characteristic suggests a new opportunity for understanding the content of biomedical images--by quantitatively exploring the spatial distribution of text information inside a biomedical image, people may gain much high-level understanding of the image, such as the image's content type. To exploit this new type of image features for content characterization, we first need to detect the presence and locations of text regions inside a biomedical image. In this work, we deploy the algorithm by Xu et al. [8] for the purpose of image text region detection and localization. Based on the spatial distribution of the detected text regions, we can then extract the aforementioned novel image features for categorizing biomedical images.

#### Entropy distribution of text regions

In our prior work [1], we have preliminarily explored the *entropy distribution of text regions *as a novel type of image feature. The entropy associated with the scanline *l_i _*can be computed according to the number of pixels encountered by the scanline across the whole input image. In our study, we empirically notice that the false recognition of image noises as foreground pixels can significantly compromise the overall accuracy of image categorization. To avoid this negative influence from image noises, we adopt a minimum foreground pixel interval threshold 5. That is, if a detected consecutive sequence of foreground pixels is shorter than 5 pixels in a row, we will discard the whole sequence as noise. Overall, image features we derive according to the entropy distribution of text regions include two 10 − *dimension *vectors $H_{\text{h,}j}^{\text{entropy}}\left(I\right)$ and $H_{\text{v}}^{\text{entropy}}\left(I\right)$, which are respectively derived by horizontally and vertically examining the entropy distributions of text regions in an input image.

#### Structural patterns regarding the spatial distributions of text regions

Another set of image features we propose to leverage is defined according to structural patterns exhibited by spatial distributions of text regions in biomedical images. Such set of features can be particularly useful and reliable for indicating biomedical images displaying structurally formated elements such as *tables *and *flow charts*. This new image feature set explores two sub-groups of structural patterns [1], which we will look at respectively in the following:

The first sub-group of image features consists of a family of five four-dimensional vectors *V*_freq,h*,j *_(*j *= 1, · · ·, 5), which describe structural patterns along the horizontal direction of an image, and another family of five four-dimensional vectors *V*_freq,v,*j *_(*j *= 1, · · ·, 5), which examine structural patterns along the vertical direction of an image. Let *ψ*_freq,h*,j *_be the set of the *j*-th Fourier coefficients derived from each horizontal scanline $l_{i}\in I$. Let *LQ*(*ψ_x_*), *Mean*(*ψ_x_*), *Median*(*ψ_x_*), and *HQ*(*ψ_x_*) respectively represent the lower quartile, mean, median, and higher quartile values of a given number set *ψ_x_*. We can then construct the two vectors mentioned in the above as follows: *V*_freq,h*,j *_= {*LQ*(*ψ*_freq,h,*j*_), *Mean*(*ψ*_freq,h*,j*_), *Median*(*ψ*_freq,h*,j*_), *HQ*(*ψ*_freq,h*,j*_)} and *V*_freq,v,*j *_= {*LQ*(*ψ*_freq,v,*j*_), *Mean*(*ψ*_freq,v,*j*_), *Median*(*ψ*_freq,v,*j*_)*, HQ*(*ψ*_freq,v,*j*_)}.

The second sub-group of image features consists of a ten-dimensional vector $H_{\text{h}}^{\text{structure}}\left(I\right)$ and another ten-dimensional vector $H_{\text{v}}^{\text{structure}}\left(I\right)$. For each horizontal scanning line $l_{i}\in I$, we derive a value denoted as *ϑ_h_*(*l_i_*), which is used for measuring the strength of the spatial structure regularity along the horizontal scanline *l_i_*. In particular, we introduce the notation *ϑ_h_*(*l_i_*, *τ*) to measure the regularity of spatial structure patterns exhibited following the horizontal scanline *l_i _*where *τ *is a threshold used to terminate the cluster merging process. For more details regarding the definition of *ϑ_h_*(*l_i_*, *τ*), readers are referred to [1]. We then further construct a ten dimensional vector $H_{\text{h}}^{\text{structure}}\left(I\right)$ by computing a ten-bin histogram that equally divides the value ranges formulated by *ϑ_h_*(*l_i_*) for $l_{i}\in I$ into ten bins. Each component $H_{\text{h,}i}^{\text{structure}}\left(I\right)$ of the histogram vector $H_{\text{h,}i}^{\text{structure}}\left(I\right)$ indicates the percentage of horizontal scanning lines that fall into the bin. Similarly, we can also derive vector $H_{\text{v}}^{\text{structure}}\left(I\right)$ by analyzing the structural distribution of text intervals along vertical scanning lines of the input image  I. For more details regarding the definition and extraction of these two groups of image features, readers are referred to an earlier preliminary publication of our work [1].

#### Distance distribution of text regions to their closest neighbours

Given a pair of text regions $<Rec_{i},Rec_{j}\in I>$, our method uses their mutual distance to indicate the distance between an arbitrary pixel on the boundary of *Rec_i _*and another arbitrary pixel on the boundary of *Rec_j_*. Let $d_{\text{max}}\left(I\right)$ be the largest distance between any pair of text regions. The method then constructs a five-band histogram, whose form is as follows: $\left[\frac{d_{\text{max}}{\left(I\right)}_{j}}{5},\frac{d_{\text{max}}\left(I\right)\left(j+1\right)}{5}\right]\;\left(j=0,\cdots \;,4\right)$. For each text region, we can calculate its distances to the *K *closest neighbouring text regions. To keep track of these distance, we introduce the vector *H*_dis_(*Rec_i_*) where its *j *− *th *component, denoted as *H*_dis*,j *_(*Rec_i_*), records the percentage of distances between *Rec_i _*and its *k *nearest neighbouring text regions that fall into the *j*-th bin of the five-band histogram constructed in the above. Following this way of construction, we can derive five sets *ψ*_dist,j _(*j *= 0, · · ·, 4) where $\psi_{\text{dist},\text{j}}≜\left\{H_{\text{dis},\text{j}}\left(Rec_{i}\right)|Rec_{i}\in I\right\}$. Furthermore, we can compute the vector $V_{\text{dis},j}≜\left\{LQ\left(\psi_{\text{dist},\text{j}}\right),Mean\left(\psi_{\text{dist},\text{j}}\right),Median\left(\psi_{\text{dist},\text{j}}\right),HQ\left(\psi_{\text{dist},\text{j}}\right)\right)$. Let *n*_Rec _be the total number of text regions involved in the above calculation. In our study, $k=\frac{n_{\text{Rec}}}{3}$ contribute a set of most discriminative features, *V*_dis*,j *_(*j *= 0, · · ·, 4), for our image categorization target.

Table 1 lists the novel image features introduced in this work for categorizing biomedical images.

**Table 1:** Summarization of our proposed novel image features

features title	features dimension
*1*. **text region entropy distribution: **$H_{\text{h}}^{\text{entropy}}\left(I\right)$, $H_{\text{v}}^{\text{entropy}}\left(I\right)$	20
*2*. **structural patterns: ***V*_freq,h,*j *_(*j *= 1, · · ·, 5), *V*_freq,v,*j *_(*j *= 1, · · ·, 5), $H_{\text{h},i}^{\text{structure}}\left(I\right)$, $H_{\text{v},i}^{\text{structure}}\left(I\right)$,	60
*3*. **distance distribution closest neighbours: ***V*_dis,*j *_(*j *= 0, · · ·, 4)	20

#### Categorizing images embedded in biomedical publications using sparse coding representation

In our work, we leverage a sparse coding based technique to categorize images embedded in biomedical publications due to its widely reported success in solving pattern categorization problems. In general, the learning method [43] considers a training set of signals **X **= [*x*_1_, · · ·, *x*_*n*_] ∈ *R*^*K* × *n *^where *K *is the total number of image features considered and *n *is the total number of training images available. The goal is to optimize the following function [43]:

(1) $$g_{n}\left(D\right)=\frac{1}{n}\overset{n}{\underset{i=1}{\sum }}f\left(x_{i},\;D\right),$$

where **D **∈ *R*^*K* × *n *^is a dictionary wherein each column represents a basis vector; *l *is a loss function such that the more precisely **D **represents the signal *x*, the smaller *f*(*x*, **D**) becomes. Under the sparse representation scheme, the loss function can be formulated as [43]:

(2) $$f\left(x,D\right)=\underset{\alpha \;\in \;R^{n}}{\text{min}}\frac{1}{2}||x-D\alpha |{|}_{2}^{2}+\lambda ||\alpha |{|}_{1},$$

where *λ *is a tradeoff parameter. This issue is considered as *basis pursuit *[44] or the *Lasso *[45]. Enforcing the penalty term ||*α *||_1 _generates a sparse solution. By employing the LARS-lasso approach [46], this kind of problem can tend to be solved efficiently. To prevent **D **from being too large, which would make the learning problem more difficult to solve with too many degrees of freedoms, it is common practice to constrain the column vectors in the dictionary *d_j _*(*j *= 1, · · ·, *n*) to have an *l*_2 _norm less than or equal to one. Adopting this constraint, we can formulate a convex set Ω of matrices as follows:

(3) $$\text{Ω}=\left\{D\in R^{K\times n},s.t.\;\;\forall_{j}=1,\cdots \;,n;d_{j}^{T}d_{j}\leq 1\right\}.$$

In our aforementioned optimization problem, the empirical cost function *g_n_*(**D**) is not convex. Instead, the problem can be reformulated as a joint optimization problem concerning variable **D **and the *α *= [*α*_1_, ..., *α_n_*] associated with the sparse decomposition. The newly formulated problem becomes convex concerning either one of the two variables **D **and *α *under the condition that the other variable among the two is fixed. Formally, we can write the new optimization objective function as (4):

(4) $$\underset{D\in \Omega ,\alpha_{i}\in \;R^{n\times 1}}{\text{min}}\frac{1}{n}\overset{n}{\underset{i=1}{\sum }}\left(\frac{1}{2}||x_{i}-D\alpha_{i}|{|}_{2}^{2}+\lambda ||\alpha_{i}|{|}_{1}\right).$$

By alternatively using the sparse coding on a given **D **to solve *α *and then updating the dictionary **D **with the derived value assignment for *α*, we can solve the problem iteratively. In our work, we employ the iterative procedure proposed by Mairal et al. [35] to obtain the optimal values of **D **and *α*.

According to Ramirez et al. [47], ideally, dictionaries corresponding to different image classes shall be as independent as possible. Assuming *X*^(*j*) ^(*j *∈ [1, *C*]) is a specific class of images and **D**^(*j*) ^is the class' corresponding dictionary. We can then state the above desired dictionary independency property as follows:

(5) $$\underset{{\left\{D^{\left(j\right)},A^{\left(j\right)}\right\}}_{j=1,\cdots ,C}}{\min}\sum_{j=1}^{C}(||X^{\left(j\right)}-D^{\left(j\right)}A^{\left(j\right)}|{|}_{2}^{2}+\lambda \sum_{r=1}^{m_{i}}||\alpha_{r}^{\left(j\right)}|{|}_{1})+\;\eta \sum_{j_{1}\neq j_{2};j_{1},j_{2}=1,\cdots \;,C}||{(D^{\left(j_{1}\right)})}^{T}D^{\left(j_{2}\right)}|{|}_{2}^{2}\;,$$

where $A^{\left(j\right)}=\left[\alpha_{1}^{\left(j\right)},\cdots \;,\alpha_{m_{i}}^{\left(j\right)}\right]\in R^{K\times m_{i}}$ in which each column vector $\alpha_{r}^{\left(j\right)}\left(r\in \left[1,\cdots \;,m_{i}\right]\right)$ is the sparse coding representation for the *r*-th image in *X*^(*j*) ^and *m_i _*is the total number of images contained in the image class *X*^(*j*)^.

#### Feature importance weights

Given a training sample image *x_i_*, we denote the image's class label as *s_i _*∈ [1, *C*]. Let *k *∈ [1, *K*] be an index variable for a specific image feature; *j *∈ [1, *C*] be a certain class label value; and *i *∈ [1, *N*] be an index variable for a specific sample in an image class. For each training example image *x_i_*, after extracting its spare coding representation, we denote the corresponding representation coefficient of *x_i _*for the *k*-th image feature and the *j*-th candidate image class as${\hat{\alpha }}_{j,i}^{k}$. We further denote the *k*-th image feature of *x_i _*as $x_{i}^{k}$. Based on the above notations, we can therefore measure the representation or reconstruction error for *x_i _*with respect to its *k*-th image feature and the *j*-th candidate image class as follows:

(6) $$R_{i,j}^{k}=\;||x_{i}^{k}-D_{k}^{\left(j\right)}{\hat{\alpha }}_{j,i}^{k}|{|}_{2}.$$

where $D_{k}^{\left(j\right)}$ is the version of the dictionary that corresponds to the *k*-th image feature of sample images belonging to the *j*-th image class. For more details regarding the dictionary derivation procedure, readers are referred to [46]. Let *ω^k ^*be the importance weight associated with the *k*-th image feature. {*ω^k^*}_*k *_= 1,··· ,*K* are the weights that measure the importance of different features in an image' overall categorization decision. In our method, all feature weights are chosen in such a way that $\sum_{k=1}^{K}\omega^{k}R_{i,s_{i}}^{k}\leq \sum_{k=1}^{K}\omega^{k}R_{i,j}^{k}-\varepsilon \;\left(\forall_{j}\neq s_{i}\right)$ where *ε *is some marginal parameter. Essentially, *ε *indicates the minimum value gap between the expression term $\sum_{k=1}^{K}\omega^{k}R_{i,j}^{k}$ when *x_i_*'s class categorization is determined correctly versus erroneously. For each *x_i_*, we further introduce a slack variable *ξ_i _*in case the above anticipated inequality does not hold in general. That is, instead of expecting $\sum_{k=1}^{K}\omega^{k}R_{i,s_{i}}^{k}\leq \sum_{k=1}^{K}\omega^{k}R_{i,j}^{k}-\varepsilon \;\;\left(\forall_{i},\forall_{j}\neq s_{i}\right)$, we now accept a more relaxed condition that $\sum_{k=1}^{K}\omega^{k}R_{i,s_{i}}^{k}-\xi_{i}\leq \sum_{k=1}^{K}\omega^{k}R_{i,j}^{k}-\varepsilon \;\;\left(\forall_{i},\forall_{j}\neq s_{i}\right)$. Putting everything together, we can formulate the overall optimization problem to determine and derive the optimal feature weight assignment {*ω^k^*} in the form of the following linear programming problem:

(7) $$\begin{array}{r} \underset{\in ,\xi ,\omega }{\min}\text{Objection Function = (}\frac{1}{N}\sum_{i=1}^{N}\xi_{i})-\varepsilon \\ s.t.\;\sum_{k=1}^{K}\omega^{k}R_{i,s_{i}}^{k}-\xi_{i}\leq \sum_{k=1}^{K}\omega^{k}R_{i,j}^{k}-\varepsilon \;(\forall i;j=1,\cdots \;,C;j\neq s_{i})\\ \sum_{k=1}^{K}\omega^{k}=1;\\ \varepsilon \geq 0;\\ \xi_{i}\geq 0,\;\omega^{k}\geq 0\;(\forall i,k). \end{array}$$

The above problem can be efficiently computed by applying a general linear programming solver. In our implementation, we adopt the solver provided by the Matlab package to derive the solution. To predict whether a target image *xi *belongs to the *j*-th candidate image class according to the image's *k*-th feature $x_{i}^{k}$, we can rely on the multiplication of the dictionary element $D_{k}^{\left(j\right)}$ and the image's corresponding sparseness parameter ${\hat{\alpha }}_{j,i}^{k}$, the value assignments for both of which are derived from the above optimization procedure. That means, we wish to approximate the value of the *k*-th image feature $x_{i}^{k}$ through $D_{k}^{\left(j\right)}{\hat{\alpha }}_{j,i}^{k}$. And the target image will be categorized into the  ĵ-th image class that maximizes such value approximations for all the features. Mathematically, this image categorization process can be stated as follows:

(8) $$ĵ=\text{arg}\underset{j\in \left[1,\;C\right]}{\text{min}}\overset{K}{\underset{k=1}{\sum }}\omega^{k}||x_{i}^{k}-D_{k}^{\left(j\right)}{\hat{\alpha }}_{j,i}^{k}|{|}_{2}^{2}.$$

In our work, all the image features considered have been introduced in the earlier part of this manuscript; so does the number of different image classes considered in the categorization task. Numerically, this setup amounts to having the number of image features *K *set to 100 and the total number of candidate image classes *C *set to 5.

## Results

In our experiments, prior to image categorization, we first introduce a new image segmentation procedure as our first step of image classification target. Once an input image is decomposed into multiple potential sub-image regions, we then extract the novel image features and apply the new image classification method both proposed in this paper. Lastly, we conducted a series of evaluation efforts to quantify the performance of the new features and categorization method.

### Image pre-processing results

To categorize biomedical images, we first need to identify a proper image taxonomy. In our work, we employ the two-level biomedical image taxonomy [1] shown in Figure 1.

To cope with images that might belong to the category of *mixed images *in our image taxonomy, before we apply the actual image categorization procedure, each input image is segmented, if multiple sub-images can be detected from the input image. As confirmed by our experimental results, the overall image categorization performance can be significantly improved by applying the image segmentation operation as a pre-processing step prior to the image categorization step. Figure 2(a) illustrates a sample image that consists of several sub-images. Figure 2(b) displays sub-images decomposed from the sample image, which consists of a lin*e chart *and a *bar chart*.

### Novel image features results

In our work, we extract three types of distribution of image text region which include: (1)improved distribution of text region entropy, (2)structural patterns of text region distribution, and (3)distance distribution closest neighbours. From Table 1 we can easy to see that the first and third groups of image features are all 20 − *dimension *vector. The second group of image features is 60 − *dimension *vector. Each image features vector consists of sub-vector of horizontal direction features and sub-vector of vertical ones.

### Categorizing experimental results

To acquire images to be used in our experimental work, we obtain all materials provided by PMC [9]by the end of year 2012. We can see some samples in Figure 3. We arbitrarily chose 990 images from our downloaded repository, among them 310 images were serve as training samples, and the rest were serve as the testing cases. We first segmented 310 sample images by using our proposed procedure. This step produced a total of 1035 sub-images. We then manually labeled all these sub-images according to image taxonomy. Among our annotation results, 316 are *microscopy images*, 126 are *gel electrophoresis images*, 135 are *line charts*, 156 are *bar charts*, 52 are *spot charts*, 25 are *tables*, 70 are *flow charts*, and the remaining 155 images are type of *others*. In our experiment, parameter *λ *is set to 0.10.

We employ standard metrics such as recall, precision, and F-score for measuring image categorization performance. Confusion matrix result first demonstrates in Table 2. From this table, we can obviously find that the numbers in diagonal line of table are the true values of categorization. Among classes of images, the class of *flow chart *owns more number of true positive image. Table 3 exhibits the performance of each class. We employ the TP, FP, and FN to measure the categorization results. TP, FP, and FN respectively respectively indicates true positive, false positive, and false negative. According to the F-scores values, this model does best on distinguishing *flow chart *class. Table 3 reveals that the class of *flow chart *still remains the good performance.

**Table 2:** Confusion matrix for our image categorization results

	Predicted categories				
	
True categories	flow chart	experiment	graph	mix	others
flow chart	136	1	5	0	4
experiment	0	82	0	12	8
graph	2	0	204	24	16
mix	0	2	4	90	4
others	1	0	3	10	63

**Table 3:** performance of image categorization using our newly proposed image features

Category	TP	FP	FN	Precision	Recall	F-score
flow chart	136	3	10	0.9784	0.9315	0.9544
experiment	82	3	20	0.9647	0.8039	0.8770
graph	204	12	42	0.9444	0.8293	0.8857
mixed	90	46	10	0.6618	0.9000	0.7627
others	63	22	14	0.7412	0.8182	0.7778

To verify our extracting novel features, we employing both traditional image features and our proposed novel features to implement the test. Traditional image feature in our experiment consists of edge-direction histogram, intensity histogram and texture. Using our proposed improved SCR categorization method, we first only use the traditional image features to conduct categorizing image task. Then, we add our novel image features with traditional image features to carry out the experiment. The results of our comparative experiments are exciting. Table 4 lists the test results. Our outcomes prevail in three indexes including *precision*, *recall*, *F-score*.

**Table 4:** Image categorization performance using the conventional image features alone versus with our novel image features

features	Precision	Recall	F-score
conventional image features	0.500	0.480	0.489
conventional image features+ **our novel image features**	0.8581	0.8567	0.8457

To more thoroughly evaluate the performance of our proposed weighted sparse coding representation (SCR), we conduct a case-control experiment where in control experimental setting, we employ the traditional SVM based method for image categorization; while in the case experimental setting, we apply our proposed weighted SCR method for image categorization. In the experimental process, we also explore the impact on image categorization performance by using different image features through a second case-control comparative setting. More specifically, in the control setting, we employ the conventional image features such as edge-direction histograms, texture-features, and intensity histograms for biomedical image categorization using the SVM method and the weighted SCR method respectively; in the case setting, we apply our proposed image features alone as well as apply both conventional image features and our new features to categorize biomedical images using the SVM method and the weighted SCR method respectively. Table 5 reports the results of the above, comprehensive, comparative studies. According to the performance numbers listed in the table, we can clearly see that our proposed SCR based image classification method coupled with the novel image features proposed in this paper can jointly achieve superior image classification accuracy to the traditional SVM based method using the same set of image features.

**Table 5:** Accuracy of image categorization achieved using different sets of image features and by the traditional SVM method versus our SCR based method

Features	SVM	SCR
conventional image features	0.65	**0.67**
**our novel image features**	**0.74**	0.73
conventional image features+ **our novel image features**	0.77	**0.846**

Table 6 indicates our method compared with peer methods (M1 [27], M2 [5], M3 [6]). We adopt accuracy to measure each method performance. Our novel features and proposed classification method are impressively effective.

**Table 6:** Comparision between our newly proposed method in this paper and three peer methods in terms of their average image categorization accuracy

Methods	Accuracy
Method 1 [27]	0.782
Method 2 [5]	0.753
Method 3 [6]	0.81
**Ours Method**	**0.846**

## Conclusions

To accurately categorize biomedical images, in this paper, we propose a novel type of biomedical image features that exploit the spatial distributions of text information inside an image for image content representation and semantics characterization. We also introduce a new weighted sparse coding based representation method for image categorization. By jointly leveraging merits of the new image features and the image categorization method, we can significantly improve the performance of biomedical image categorization task, whose effectiveness is confirmed by the results of all our experimental results. According to the results of comparative studies reported in Table 6 it is clear to see that the performance produced by our new method has significantly outperformed all existing methods based on traditional image features and conventional image categorization methods. Our improved image categorization approach can benefit many applications in retrieving and content mining biomedical images.

Our method is designed for categorizing biomedical images, for which we notice that one of the salient characteristics of such images is their abundant embedded text information. By exploring and quantifying the spatial distributions of these text elements embedded inside an image, we can effectively boost the performance of image categorization. We, however, notice that for more common categories of images, which only carry sparse or no text information, the new image features proposed in this paper do not possess advantages than the traditional features. In our future research, we plan to investigate dynamic feature selection and weighted classification method to adaptively engage proper types of image features for categorizing images in a broader domain.

## Competing interests

The authors declare that they have no competing interests.

## Authors' contributions

Sheng proposed the image categorization method and conducted the experiments. Xu proposed the novel image features used in this paper, directed the whole research effort, and prepared the manuscript. Sheng and Xu also jointly performed data analysis, interpreted the results, and drafted the manuscript. Luo provided the experiment platform and supported the paper works. All authors read and approved the final manuscript.

## Declarations

The publication costs for this article were funded by the corresponding author.

This article has been published as part of *BMC Medical Genomics *Volume 6 Supplement 3, 2013: Selected articles from the IEEE International Conference on Bioinformatics and Biomedicine 2012: Medical Genomics. The full contents of the supplement are available online at http://www.biomedcentral.com/bmcmedgenomics/supplements/6/S3.
